# Assessing the Readiness to Provide Integrated Management of Cardiovascular Diseases and Type 2 Diabetes in Kenya: Results from a National Survey

**DOI:** 10.5334/gh.1213

**Published:** 2023-06-15

**Authors:** Peter Otieno, Charles Agyemang, Welcome Wami, Calistus Wilunda, Richard E. Sanya, Gershim Asiki

**Affiliations:** 1 Department of Public & Occupational Health, Amsterdam UMC, University of Amsterdam, Amsterdam Public Health Research Institute, Amsterdam, The Netherlands; 2 African Population and Health Research Center P.O. Box: 10787-00100, Nairobi, Kenya; 3 Amsterdam Institute for Global Health and Development (AIGHD), AHTC, Tower C4, The Netherlands; 4 Department of Women’s and Children’s Health, Karolinska Institutet, Stockholm, Sweden

**Keywords:** integrated care, cardiovascular diseases, type 2 diabetes, readiness assessment

## Abstract

**Introduction:**

Integrated chronic disease management is the desired core function of a responsive healthcare system. However, many challenges surround its implementation in Sub-Saharan Africa. The current study assessed the readiness of healthcare facilities to provide integrated management of cardiovascular diseases (CVDs) and type 2 diabetes in Kenya.

**Methods:**

We used data from a nationally representative cross-sectional survey of 258 public and private health facilities conducted in Kenya between 2019 and 2020. Data were collected using a standardised facility assessment questionnaire and observation checklists modified from the World Health Organization Package of Essential Non-communicable Diseases. The primary outcome was the readiness to provide integrated care for CVDs and diabetes—defined as the mean availability of tracer items comprising trained staff and clinical guidelines, diagnostic equipment, essential medicines, diagnosis, treatment and follow-up. A cut-off threshold of ≥70% was used to classify facilities as ‘ready’. Gardner-Altman plots and modified Poisson regression were used to examine the facility characteristics associated with care integration readiness.

**Results:**

Of the surveyed facilities, only a quarter (24.1%) were ready to provide integrated care for CVDs and type 2 diabetes. Care integration readiness was lower in public versus private facilities [aPR = 0.6; 95% CI 0.4 to 0.9], and primary healthcare facilities were less likely to be ready compared to hospitals [aPR = 0.2; 95% CI 0.1 to 0.4]. Facilities located in Central Kenya [aPR = 0.3; 95% CI 0.1 to 0.9], and the Rift Valley region [aPR = 0.4; 95% CI 0.1 to 0.9], were less likely to be ready compared to the capital Nairobi.

**Conclusions:**

There are gaps in the readiness of healthcare facilities particularly primary healthcare facilities in Kenya to provide integrated care services for CVDs and diabetes. Our findings inform the review of current supply-side interventions for integrated management of CVDs and type 2 diabetes, especially in lower-level public health facilities in Kenya.

## Background

Cardiovascular diseases (CVDs) and type 2 diabetes are the leading contributors to the global burden of morbidity and mortality [1,2]. Three-quarters of CVDs and diabetes-related mortalities occur in low and middle-income countries (LMICs) [3]. In Kenya, CVDs are responsible for 50% of hospital admissions and 40% of hospital mortalities [4]. Complications of hypertension remain the most prevalent among all CVDs [5]. One in four adults in Kenya has hypertension, and 3% have type 2 diabetes [6,7]. Half of the patients with type 2 diabetes in Kenya also have hypertension [8]. From the 2015 nationwide non-communicable diseases (NCD) STEPS survey, only 15.6% and 43.7% of people living with hypertension and diabetes were aware that they had hypertension and type 2 diabetes, respectively [6,7]. Only 26.9% of individuals with hypertension and 20.0% of those with type 2 diabetes were on treatment, with more than half of those on treatment having poor control [6,7].

The rising global burden of CVDs and type 2 diabetes requires a shift from vertical disease management programs toward integrated care [9]. Healthcare services are ‘integrated’ when services for two or more diseases are offered at the same facility during the same visit, and the provider of one service encourages patients to consider using the other service during the visit [10]. Previous studies show that access to integrated healthcare services has the potential to elicit positive health outcomes, including improved prognosis, clinical outcomes, and overall quality of life [11,12,13,14,15]. However, many challenges surround its design and implementation [16,17]. People living with CVDs and type 2 diabetes often have multimorbidities (multiple conditions) [18]. In 2019, about one-third of adults in the world lived with multimorbidities, typically including CVDs and type 2 diabetes [19].

The United Nation’s sustainable development goal of universal health coverage envisages access to high-quality integrated healthcare services [20]. Kenya has set out several measures to curb the burden of CVDs and diabetes [21,22,23]. At the policy level, chronic disease prevention and management has been prioritised as one of the key objectives of the Kenya Health Policy 2014–2030 [24]. Kenya established national guidelines for preventing and managing diabetes and CVDs in 2010 and 2018, respectively [25,26]. These guidelines follow the WHO HEARTS technical package for CVD management in primary health care [25,27]. One of the major policy directions toward addressing the burden of CVD and type 2 diabetes is to provide integrated care [25]. Consequently, the Kenya Expanded Program on Health has included care for CVDs and type 2 diabetes in the essential package of primary healthcare [28].

Despite these policy initiatives, little is known about the extent to which the healthcare facilities in Kenya apply the elements of care integration envisioned in the national policy guidelines. A readiness assessment of care integration capacity is crucial for benchmarking the health system response to the burden of CVDs and type 2 diabetes and supporting policy-makers in planning sustainable chronic care models. To respond to this need, this study assessed the readiness of public and private healthcare facilities in Kenya to provide integrated management of CVDs and type 2 diabetes.

## Methods

### Study design and setting

We analysed secondary data from a nationally representative cross-sectional study conducted between 2019 and 2020 investigating Kenya’s healthcare system response to chronic disease management [29]. The overarching aim of the study was to strengthen the health system’s responsiveness to managing chronic diseases in Kenya. The health service delivery in Kenya is structured in a six-tiered system ranging from levels 1 to 6 [30]. Level 1 comprises community services with no physical infrastructure while level 2 are small clinics and dispensaries. Level 3 consists of small maternity clinics and community health centres. The sub-county hospitals and the county teaching and referral hospitals are classified as levels 4 and 5, respectively, while level 6 includes the national teaching and referral hospitals. The county governments are responsible for the first five levels of care while the national government is responsible for the sixth level.

### Sample size

The process of sample size estimation is explained in detail elsewhere [29]. The sample size for the study was calculated using the formula commonly used for Service Availability and Readiness Assessment Mapping (SARAM) surveys. The SARAM surveys are nationally representative systematic surveys designed by the WHO to generate reliable and regular information on a set of tracer indicators of service availability and readiness [31]. These include the availability of human resources, infrastructure, equipment, essential medicines, diagnostic capacities and the readiness of health facilities to provide primary healthcare interventions for infectious and non-communicable diseases [31]. Based on a pilot study conducted in Machakos and Nairobi counties in 2016–2017, 40% of the facilities in Kenya were assumed to be ready to deliver some aspects of chronic disease management [23]. A design effect of 1.2 and 15% margin of error were used in the sample size calculation, with adjustment for a non-response rate of 10%. Thus, the final sample was 301 health facilities [29].

### Selection of facilities

**Figure 1:**
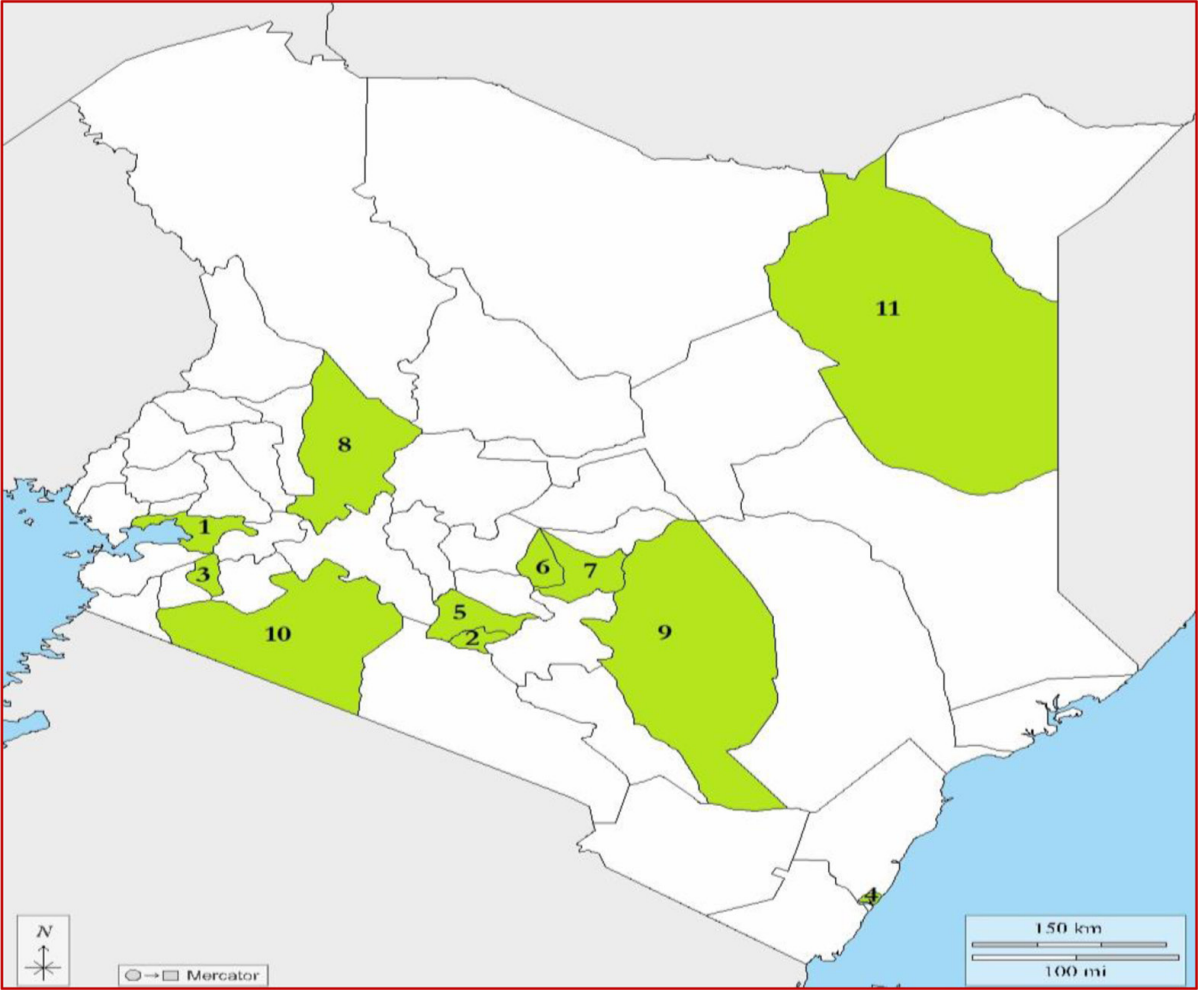
A map of Kenya showing the counties included in the health facility survey. 1 = Kisumu, 2 = Nairobi, 3 = Nyamira, 4 = Mombasa, 5 = Kiambu, 6 = Kirinyaga, 7 = Embu, 8 = Baringo, 9 = Kitui, 10 = Narok, 11 = Wajir. Blank map retrieved and adapted from: https://d-maps.com/ [Accessed: 16 May 2022].

Figure 1 shows the geographical distribution of the study counties. The study facilities were randomly selected using a multistage stratified sampling method. Kenya was first stratified into six geo-political regions: Nairobi, Central, Coast and North-Eastern, Eastern, Nyanza and Western, and Rift Valley. In the first stage, two counties (sub-counties in the case of Nairobi) were randomly sampled in each region by probability proportional to size, with size being the total number of healthcare facilities in the county. The 12 randomly selected study counties comprised Kisumu, Nyamira, Mombasa, Wajir, Baringo, Narok, Kitui, Embu, Kirinyaga, Kiambu and two sub-counties in Nairobi (Dagoretti and Starehe). The second stage involved sampling healthcare facilities in each county. A sampling frame consisting of healthcare facilities in each county was drawn from the Kenya Health Master Facility List of 2019 [32]. The healthcare facilities were stratified by levels (levels 2 to 6) and type of ownership (private or public). Stratified simple random sampling was then used to select 301 healthcare facilities from the 12 study counties. However, data were successfully collected in 258 out of the 301 sampled facilities (response rate of 86%).

### Data collection

Data were collected using an interviewer-administered structured facility assessment questionnaire and observation checklists modified from the WHO Package of Essential Non-communicable Disease Interventions for Primary Health Care (WHO-PEN). The respondents were facility in-charges and heads of clinical departments. In some higher-level facilities, more than one health service provider participated in the study because some of the tracer items assessed were located in different departments. The data collected comprised facility characteristics including the level of care (levels 2 to 6), type of managing authority (private or public), setting (urban or rural) and region in Kenya. Other data comprised equipment, service availability, human resources, clinical guidelines, essential medicines, medical record system, nutrition monitoring and self-management support. The interview responses were confirmed by direct observation using a checklist for all services where it was potentially feasible. Details of the measurements of the variables used in the current analysis are shown in Table 1.

**Table 1:** Tracer indicator items for service availability and readiness.


DOMAINS	RESOURCES

Trained staff, and clinical guidelines,	Trained staff on cardiovascular disease diagnosis and treatment Trained staff on diabetes diagnosis and treatment Clinical guidelines for the management of cardiovascular diseases Clinical guidelines for the management of diabetes

Basic diagnostic equipment	Blood pressure apparatus Weighing machine Stethoscope Blood glucose test Urine dipstick-protein Urine dipstick-ketones Blood analyzer for cholesterol screening (Level 3–6) facilities Electrocardiogram (ECG) (Level 3–6) facilities X-ray machine (Level 3–6) facilities

Essential medicines	ACE inhibitor (enalapril) Thiazide Beta-blocker (atenolol) Calcium channel blocker (amlodipine) Aspirin (acetylsalicylic acid) capsules/tablets Metformin capsules/tablets Hydrochlorothiazide tablet or other thiazide diuretic tablet Furosemide Statins Aspirin Glibenclamide capsules/tablets Insulin regular injectable Gliclazide tablet or glipizide tablet

Diagnosis, treatment, follow-up & self-management support	Diagnosis & treatment of cardiovascular diseases Diagnosis & treatment of diabetes Referral services Nutrition monitoring services Counseling services on lifestyle risk factors Hypertension and diabetes self-management counselling Electronic medical records systems on patient information, symptoms, examination, diagnosis and prescription


### Definition of variables

The outcome variable was the readiness of a healthcare facility to provide integrated care services for CVD and type 2 diabetes. In line with the WHO [33], we defined and operationalised integration readiness as ‘a one-stop center’ with essential resources for comprehensive management of CVDs and type 2 diabetes following the Kenya national guidelines for CVDs and diabetes management requirements for levels 2 to 6 facilities [25]. Integration readiness requires a trained workforce, essential drug supplies, treatment and diagnostic resources and structural improvements to support comprehensive management of CVDs and diabetes [33,34]. Hence, the assessment for care integration capacity and readiness in the present study was based on the availability of tracer items across four domains: trained staff and clinical guidelines, basic diagnostic equipment, essential medicines, diagnosis, treatment and follow-up. Details of the tracer items for each domain is shown in Table 1. For each of domain, we calculated an index as the mean score of items expressed as a percentage. The facility readiness index was then calculated as the average of domain indices expressed as a percentage. Service readiness scores ranging from 70% to 100% reflect better preparedness [35]. Hence, health facilities that scored ≥70% were classified as ‘ready’. The explanatory variables included facility characteristics: level of care, type of managing authority (private or public), setting (urban or rural) and region in Kenya.

### Data analysis

#### Sample weights

The sample facilities were weighted using the probability of selection at each sampling stage. The sampling strata comprised region, county, healthcare level and facility type. Thus, a facility’s weight was equal to the inverse of the product of the probability of the selection at each sampling stage.

#### Descriptive analysis

Descriptive statistics comprising frequencies and percentages or means and standard deviations were used to summarise the characteristics of the study facilities. We used the Gardner-Altman estimation plots to determine the differences in the mean care integration readiness by facility characteristic [36,37]. The biases in the confidence intervals of the Gardner-Altman estimation plots were corrected using 5,000 accelerated bootstraps resamples [26].

#### Regression analysis

Modified Poisson regression analysis with robust error variances was used to determine the association of care integration capacity and readiness with facility characteristics. Generally, since using a P-value threshold to select variables can fail to identify important covariates [38], we included all the facility characteristics in the multivariable model. Bayesian Information criterion was used to determine changes in the overall fit of the bivariable and multivariable models. The strength of association was assessed using adjusted prevalence rate ratios (PR) and 95% confidence intervals (CI).

Data analysis was carried out using Stata version 17 (StataCorp, College Station, TX, USA). Estimation statistics and the Gardner-Altman plots [36] were performed using R Statistical Software (version 4.1.0; R Foundation for Statistical Computing, Vienna, Austria).

## Results

### Health facility profiles

The health facility characteristics are presented in Table 2. Most facilities were level 2 (54.6%) and 3 (34.1%), publicly owned (67.8%), and located in urban settings (53.5%). In addition, three of the five national hospitals (level 6) in Kenya were included in the sample. The facilities were proportionately distributed across the six geo-political regions in Kenya (Nairobi, Central, Coast and North-Eastern, Eastern, Nyanza and Western, and Rift Valley).

**Table 2:** Health facilities profiles.


CHARACTERISTICS	N = 258

Level	n (%)

Level 2	140 [54.3]

Level 3	88 [34.1]

Level 4	19 [7.4]

Level 5	8 [3.1]

Level 6	3 [1.2]

Type	

Private	83 [32.2]

Public	175 [67.8]

Setting	

Urban	138 [53.5]

Rural	120 [46.5]

Region	

Central	39 [15.1]

Coast and North-Eastern	45 [17.4]

Eastern	45 [17.4]

Nairobi	36 [14.0]

Rift Valley	46 [17.8]

Western and Nyanza	47 [18.2]


### Healthcare services availability and readiness

**Figure 2:**
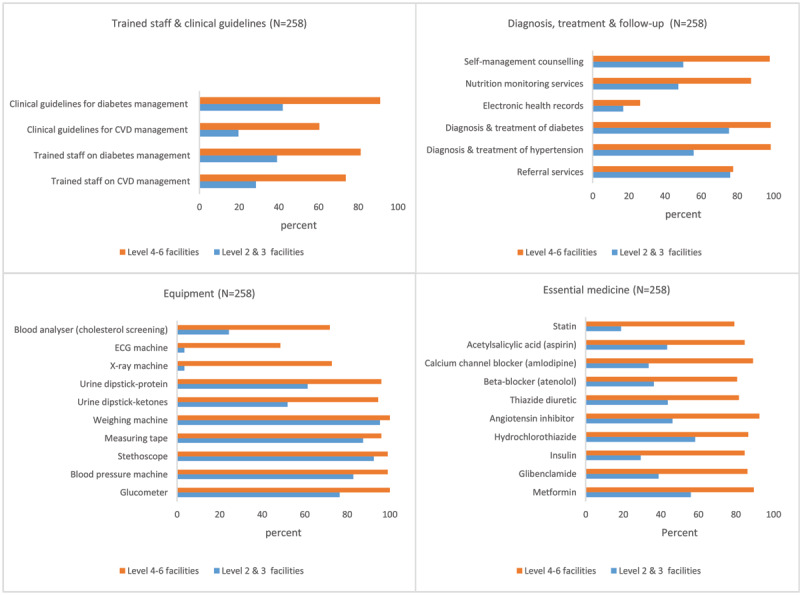
Healthcare services availability for management of CVD and type 2 diabetes.

The availability of tracer items for the management of CVDs and diabetes is shown in Figure 2. Trained staff, clinical guidelines and essential medicines were generally unavailable in most level 2 and 3 facilities but available in level 4 to 6 facilities. Under the domain of basic equipment, weighing machines, measuring tapes, stethoscopes, glucometers and blood pressure machines were generally available across most facilities. Urine dipsticks for ketones and proteins were available in most level 4–6 facilities (94.6% and 96.0%), respectively, and only available in 51.9% and 61.4% of level 2 and 3 facilities, respectively. Blood analysers were available in a quarter of level 2 and 3 facilities compared to 71.8% in level 4 to 6 facilities. X-ray and ECG machines were only available in 3.4% of level 2 and 3 facilities compared to 72.8%, and 48.5% in level 4 to 6 facilities. Diagnosis, treatment, referral and preventive services for CVD and diabetes were generally available across all the facilities except hypertension diagnosis and treatment, which was only available in 58.9% of level 2 and 3 facilities compared to 98.5% of level 4–6 facilities. Electronic health records systems were available in 26.4% of level 2–3 facilities and 16.9% of level 4–6 facilities.

### Care integration readiness for CVD and type 2 diabetes

**Figure 3:**
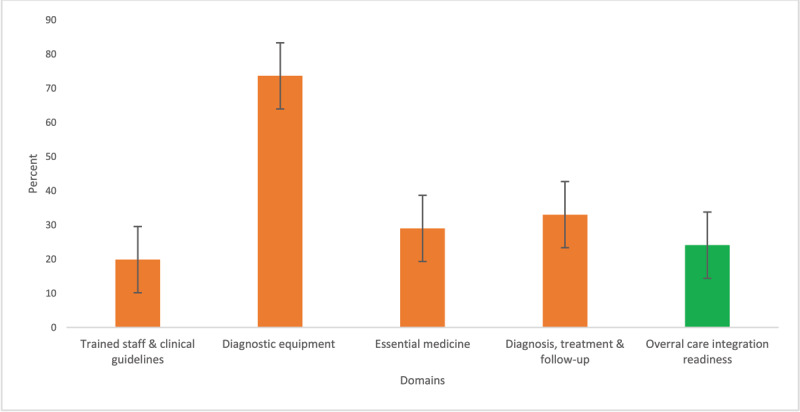
Care integration readiness for management CVD and type 2 diabetes.

The average domain score for care integration readiness items is shown in Figure 3. Overall, only a quarter (24.1%) of the healthcare facilities were ready to provide integrated care for CVDs and type 2 diabetes. About three-quarters of the facilities had diagnostic equipment for CVDs and diabetes while less than a third had the minimum threshold for ‘trained staff and clinical guidelines’, ‘equipment’, ‘essential medicines’ and ‘diagnosis, treatment and follow-up’.

### Comparison of care integration readiness scores and facility characteristics

**Figure 4:**
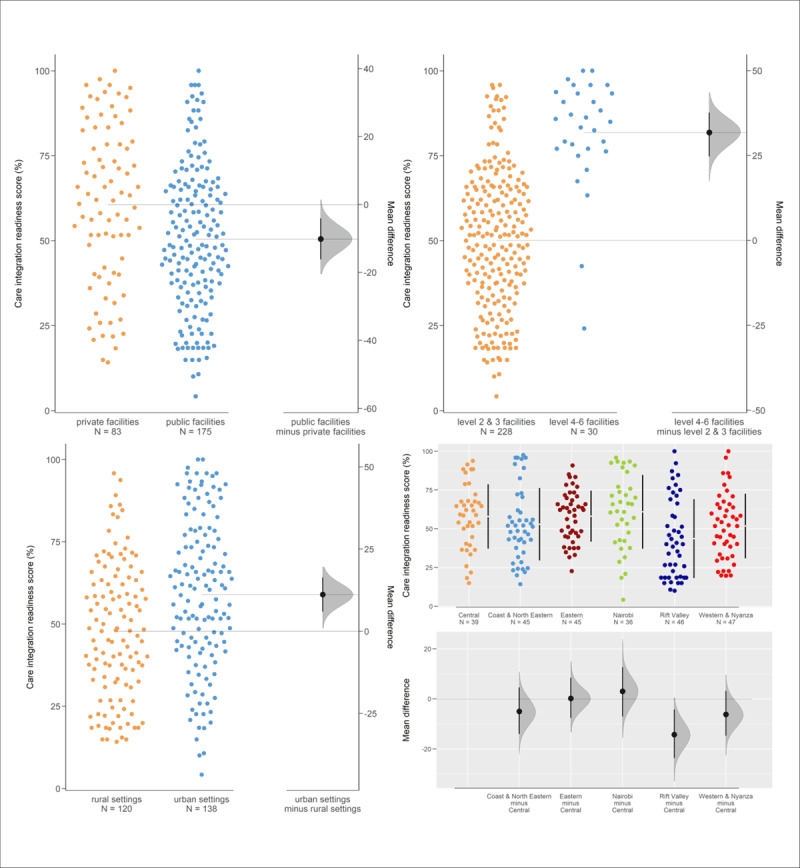
Gardner-Altman comparisons plots of care integration readiness scores and facility characteristics.

The Gardner-Altman comparisons plots of care integration readiness scores and facility characteristics is shown in Figure 4. The mean difference and 95% CI are represented by the bold black dot and line, respectively. The results show significant differences in the mean care integration readiness index across facility type, level of care, rural-urban settings and geographical regions. Level 4–6 facilities, private facilities and healthcare facilities in urban settings had higher mean care integration readiness index than level 2 and 3 facilities, public facilities and healthcare facilities in rural settings, respectively. Similar patterns were observed across regions. The mean care integration readiness index was highest in Nairobi and lowest in Rift Valley. The magnitude of the facility level differences in the mean care integration readiness index was higher compared to those observed by type of facility, rural-urban setting and geographical region.

### Facility characteristics associated with CVD and type 2 diabetes care integration readiness

**Figure 5:**
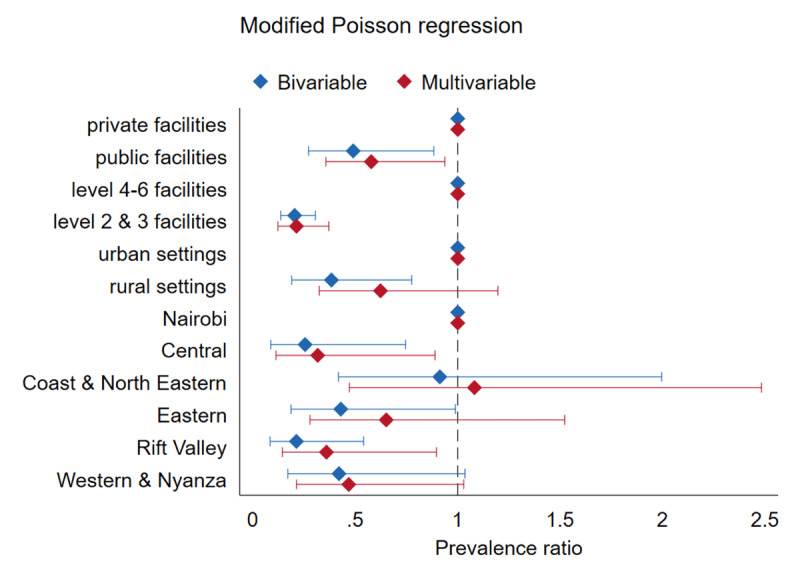
Facility characteristics associated with CVDs and type 2 diabetes care integration readiness.

The results of the modified Poisson regression are shown in Figure 5. In the adjusted model, care integration readiness was lower in public versus private facilities [aPR = 0.6; 95% CI 0.4 to 0.9], and primary healthcare facilities were less likely to be ready compared to hospitals [aPR = 0.2; 95% CI 0.1 to 0.4]. Facilities located in Central Kenya [aPR = 0.3; 95% CI 0.1 to 0.9], and the Rift Valley region [aPR = 0.4; 95% CI 0.1 to 0.9], were less likely to be ready compared to the capital Nairobi.

## Discussion

We examined the readiness of health facilities to provide integrated management of CVDs and type 2 diabetes and the associated facility characteristics in Kenya. Overall, only a quarter of the healthcare facilities were ready to provide integrated care for CVDs and type 2 diabetes. Care integration readiness was lower in public versus private facilities, level 2 and 3 versus level 4–6 facilities and facilities located in the study counties in Central and Rift Valley regions versus Nairobi. Most of the facilities included in the current study failed to reach the minimum threshold for ‘trained staff and clinical guidelines’, ‘essential medicines’, and ‘diagnosis, treatment and follow-up’. However, basic medical equipment including weighing machines, measuring tapes, blood pressure machines, stethoscopes and glucometers were generally available in most facilities.

Our findings show gaps in the readiness of Kenyan healthcare facilities to provide integrated care services for CVDs and type 2 diabetes. This finding aligns with previous studies from Kenya and other LMICs [39,40,41,42,43]. The 2013 SARAM study conducted in Kenya also found that only a third of healthcare facilities were ready to manage (NCDs) [44]. Other SARAM surveys performed in sub-Saharan African countries showed that less than half of healthcare facilities were ready to manage type 2 diabetes and CVDs in Sierra Leone, Tanzania, Zambia and Uganda [45,46,47,48]. However, it is important to note that the definition of readiness in the SARAM surveys differed from the one used in our study. The SARAM surveys take into account single morbidities [49], while the present study focused on care integration readiness for CVDs and type 2 diabetes. The gaps in the availability of integrated healthcare services for CVDs and type 2 diabetes in our study may partly explain the high burden of unmet needs for hypertension and type 2 diabetes in Kenya [6,7].

Our results indicate progress in the availability of basic diagnostic equipment, as the 2008–2012 strategic report by the Ministry of Public Health and Sanitation outlined a general lack of basic diagnostic equipment to support healthcare service delivery for NCDs in Kenya [50]. The shortage of trained staff and essential medicines for diabetes and CVDs in the current study is in tandem with the SARAM survey conducted in Kenya in 2013 [44]. Indeed, shortage of medication, trained staff and clinical guidelines are among the major health system challenges affecting the control of CVDs and diabetes in LMICs [51,52]. In, Kenya less than a quarter of people on medication for hypertension and diabetes are controlled [6,7]. The high burden of uncontrolled hypertension and diabetes could be partly due to the non-availability of medicines. Consistent with previous studies conducted in SSA [53,54], our results show low uptake of the treatment guidelines for diabetes and CVDs, particularly at a primary healthcare level, where most patients with diabetes and CVDs are likely to seek care. In particular, the shortage of physicians in most primary healthcare settings in Kenya [55], calls for new task-shifting strategies to improve the training of nurses and clinical officers on the treatment guidelines for diabetes CVDs.

Similar to previous studies [56,57,58], our results show significant disparities in CVDs and type 2 diabetes care integration readiness across facility types, levels of care and geographical settings. The disparities in the care integration readiness across facility levels were not unexpected, as the Kenyan health system is deliberately structured in a tier system with varying levels of care, from level 2 (small clinics and dispensaries) to level 6 (national referral hospitals) [59,60]. However, the substantial variation found within facility types could be partly due to the fact that private health facilities may be more efficient and responsive to patient needs than public facilities [61]. A possible explanation for the observed regional differences in care integration readiness for CVDs and type 2 diabetes may be due to, in part, the rapid urbanisation in the capital Nairobi that has increased the influx of people and high concentration of human resources for health, thereby creating the need to expand and improve health services for chronic diseases [62]. However, the healthcare service delivery for diabetes and CVDs in facilities located the study counties in Rift valley and Central region may not have received the needed attention. Similar findings of regional disparities in healthcare service readiness for chronic disease have been reported in Ghana, Nigeria and Nepal [63,64,65].

Overall, this study has two key policy implications for the integrated management of CVDs and type 2 diabetes in Kenya and extends to other countries in SSA. First, the results point to gaps in implementing the national guidelines for managing CVDs and type 2 diabetes in Kenya. The widespread unavailability of essential resources and capacity in most primary care facilities exemplifies this gap. Incorporating these findings into programmatic interventions and resource allocations may improve equity in access to healthcare services for CVDs and type 2 diabetes. Second, the service availability and readiness for the management of CVDs and type 2 diabetes were remarkably heterogeneous across facility types, locations, and ownership, and this phenomenon is likely to evolve. This highlights the need for continuous and timely assessment of the health system’s capacity and readiness for timely identification of gaps and areas of successful implementation.

### Strengths and limitations

This study assessed the capacity and readiness to provide integrated management of CVDs and type 2 diabetes in a nationally representative sample of public and private health facilities in Kenya, which increases the generalisability of the findings in the country. The data were collected using a standardised facility assessment questionnaire and observation checklists modified from the WHO Package of Essential Noncommunicable Disease Interventions for primary healthcare. Therefore, the results are comparable to those of other studies conducted in LMICs.

The results of this study should be interpreted in light of a few limitations. First, some of the domain tracer items used in the assessment of care integration capacity and readiness could not be verified by visual observation. Hence, there is a possibility of information bias and underestimation of the gaps. Second, this being a cross-sectional study means causality cannot be inferred. Lastly, the care integration capacity and readiness indicator was constructed without taking into account the patient-perceived perspectives on the quality of healthcare services. We acknowledge the importance of patient perceptions on the quality of healthcare services [66]. However, studies have shown a higher likelihood of social-desirability bias from perceived quality than objective assessments [67,68,69]. Despite these limitations, the findings of this study provide crucial insights on the overall readiness of Kenya’s healthcare system to provide integrated management of CVDs and type 2 diabetes and identified gaps that require targeted interventions.

## Conclusions

This study provides an in-depth assessment of health facility capacity and readiness for integrated management of CVDs and diabetes. The findings indicate that Kenya is far behind in being ready to provide integrated management of CVDs and type 2 diabetes, especially in public facilities and lower-level tiers. This evidence could potentially inform the review of current supply-side interventions for integrated management of CVDs and type 2 diabetes. The findings highlight the need for multifaceted primary care strengthening approaches for improving the equitable supply of essential equipment and first-line medicines, guidelines, counselling and education for patients, and regular training of healthcare workers in lower lower-level facilities. Further studies on the health system facilitators and barriers to the integrated management of CVDs and type 2 diabetes are needed to inform the design of contextually appropriate care models in Kenya.

## Data Accessibility Statement

The datasets used in this study are available upon a reasonable request to the African Population and Health Research Center (APHRC) through its Microdata portal (https://microdataportal.aphrc.org/index.php/catalog/124).

## Abbreviations

aPR: adjusted prevalence ratio; CI: confidence interval; CVD: cardiovascular diseases; ECG: electrocardiogram; LMIC: low and middle-income countries; SARAM: Service Availability and Readiness Assessment Mapping; WHO: World Health Organization; WHO-PEN: World Health Organization Package of Essential Non-communicable Diseases.
